# Robotic uterine transposition and reposition in a patient with rectal cancer: complete case report and surgical perspective

**DOI:** 10.1093/jscr/rjag403

**Published:** 2026-05-29

**Authors:** Mona P Roshan, Karl Sayegh, Eric D Schroeder, John P Diaz, Jean-Marie Stephan

**Affiliations:** Herbert Wertheim College of Medicine, Florida International University, Miami, FL, United States; Herbert Wertheim College of Medicine, Florida International University, Miami, FL, United States; Baptist Health of South Florida, Miami, FL, United States; Herbert Wertheim College of Medicine, Florida International University, Miami, FL, United States; Baptist Health of South Florida, Miami, FL, United States; Herbert Wertheim College of Medicine, Florida International University, Miami, FL, United States; Baptist Health of South Florida, Miami, FL, United States; Herbert Wertheim College of Medicine, Florida International University, Miami, FL, United States; Baptist Health of South Florida, Miami, FL, United States

**Keywords:** rectal cancer, fertility preservation, uterine transposition, uterine reposition

## Abstract

Uterine transposition (UT) is an emerging fertility-preserving technique for women requiring pelvic radiation. We report our first complete robotic UT followed by successful robotic uterovaginal anastomosis (uterine reposition, UR) in the multimodal treatment of rectal adenocarcinoma. A 29-year-old nulligravid woman with locally advanced rectal cancer underwent robotic UT in February 2024 prior to neoadjuvant chemoradiation. The uterus and adnexa were transposed to the upper abdomen with preservation of vascular supply, perfusion confirmed by fluorescence angiography, and a temporary neocervical canal created at the umbilicus. In March 2025, robotic UR was performed concurrently with abdominoperineal resection and end colostomy. Surgery was completed without intraoperative complications. Pathology revealed mucinous adenocarcinoma with one positive lymph node and negative margins. Despite later systemic recurrence, the uterus remained viable and menstruation resumed through the native cervix. This case demonstrates the feasibility of robotic UT and UR for fertility preservation in complex oncologic care.

## Introduction

Pelvic radiation can irreversibly impair both ovarian and uterine function, limiting reproductive potential beyond what ovarian preservation alone can achieve [1, 2]. Uterine transposition (UT), first described by Ribeiro *et al.* in 2017, has since emerged as a novel fertility-preserving approach for women requiring pelvic radiation [3]. While feasibility has been established and isolated pregnancies reported, most published cases describe only the transposition phase [4–7]. Reports of successful uterine repositioning (URing) are limited, and none have detailed a full robotic UT-UR cycle in the United States [8, 9].

We present the case of a young woman with rectal adenocarcinoma who underwent robotic UT prior to chemoradiation and, after completion of oncologic therapy, robotic uterovaginal anastomosis with uterine take-down and return to the pelvis.

## Case presentation

### Stage 1: Uterine transposition

**Figure 1:**
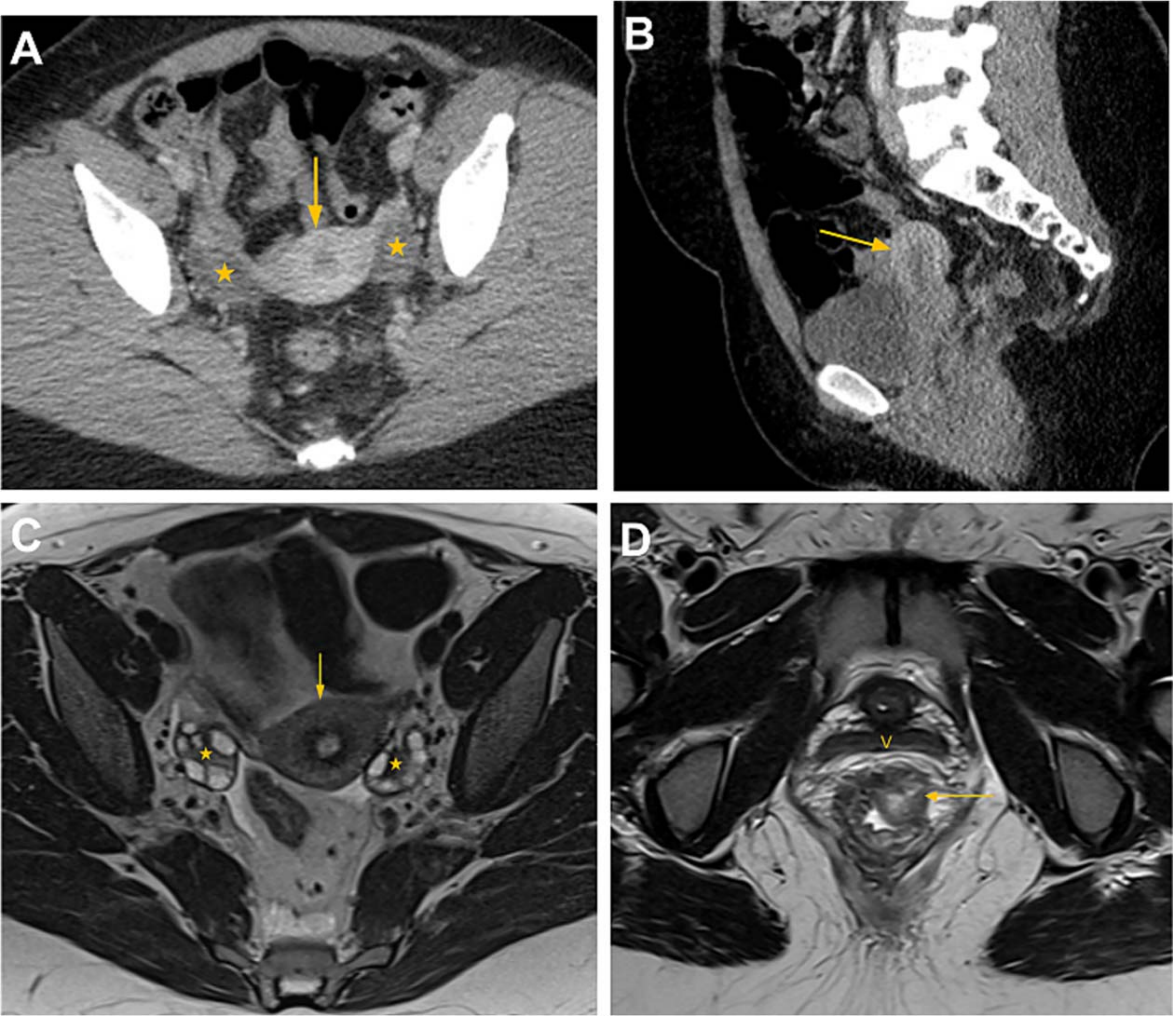
Computed tomography (CT) and magnetic resonance imaging (MRI) of the pelvis prior to uterine transplantation. Axial (A) and sagittal (B) contrast-enhanced CT images of the pelvis demonstrate the uterus (arrow) and bilateral ovaries (*) in normal position. (C) Axial T2-weighted MRI image shows normal pelvic anatomy with the uterus (arrow) and ovaries (*) containing physiological follicles. (D) Axial T2-weighted MRI image at the level of the vagina (V) demonstrates a 1.8 × 2.1 cm polypoid, heterogeneous lesion involving the left anterolateral aspect of the distal rectal wall, consistent with adenocarcinoma (arrow).

**Figure 2:**
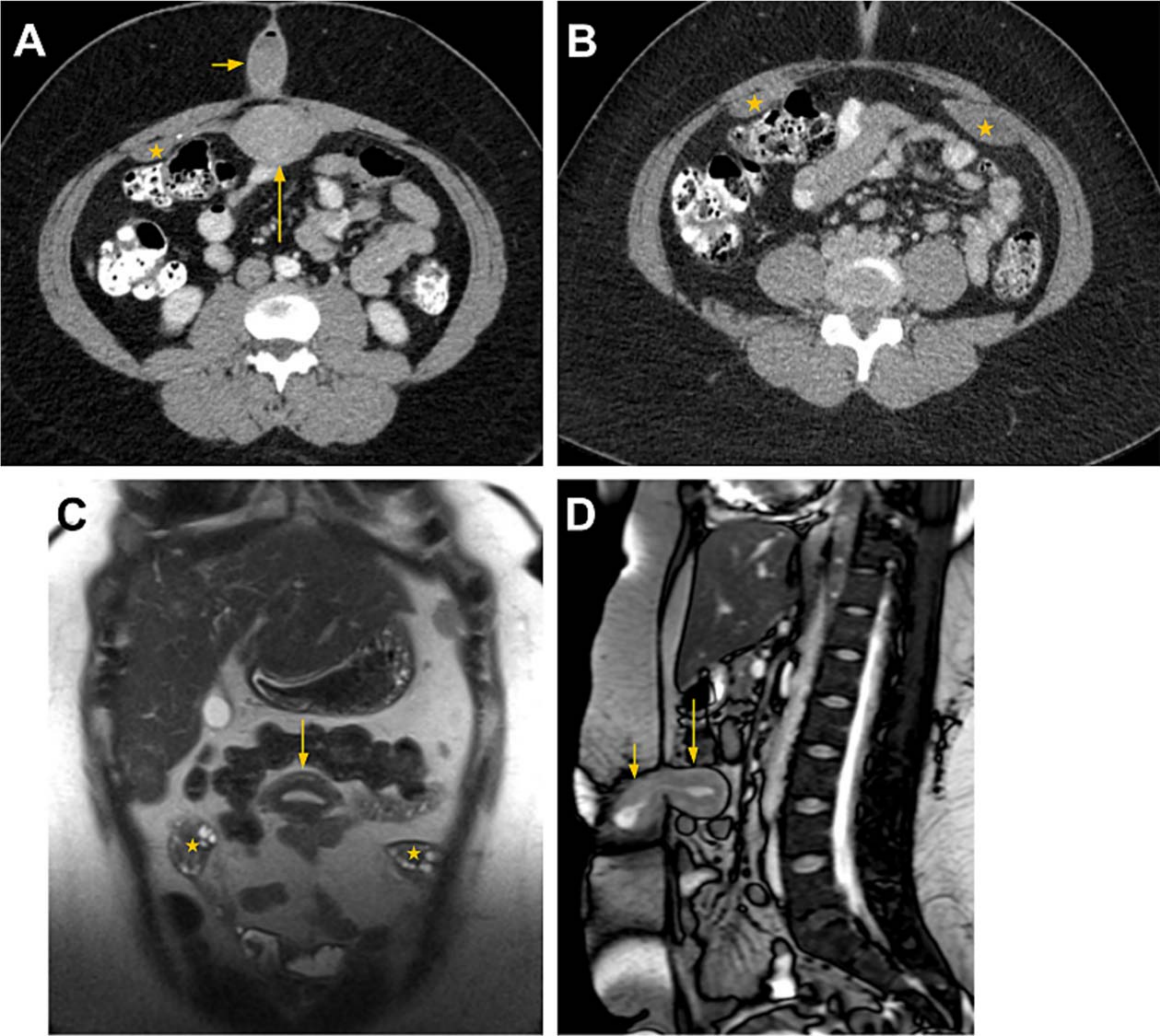
CT and MRI findings after uterine transposition. (A, B) Axial CT images of the abdomen and pelvis at the level of the umbilicus following intravenous and oral iodinated contrast administration show the transposed uterus (long arrow in A) located in the anterior midline abdomen and the neocervical umbilical canal containing a small amount of fluid and minimal air (short arrow in A). The bilateral ovaries have also been transposed and appear as intraperitoneal, featureless structures along the anterior abdominal wall (* in A and B). (C) Coronal T2-weighted MRI image demonstrating the intraabdominal, intraperitoneal position of the uterus (arrow in C) and bilateral ovaries (* in C). The characteristic uterine zonal anatomy-outer myometrium with intermediate T2 signal, junctional zone with low T2 signal, and endometrium with high T2 signal-helps differentiate the uterus from adjacent bowel loops. Physiologic ovarian cysts, when present, assist in identifying the intraabdominal ovaries. (D) Sagittal gradient-echo MRI image showing the intraabdominal, intraperitoneal uterus (long arrow in D) and the cervix (short arrow in D) within the umbilical canal.

The patient initially underwent robotic-assisted UT before neoadjuvant chemoradiation. The uterus and adnexa were mobilized and transposed to the anterior abdominal wall with preservation of vascular supply (Figs 1 and 2). Indocyanine greend (ICG) fluorescence confirmed perfusion. A neocervical canal was fashioned at the umbilicus for menstrual drainage. Recovery was uncomplicated, she resumed menstruation and completed neoadjuvant chemoradiotherapy successfully.

### Stage 2: UR and concurrent colorectal surgery

**Figure 3:**
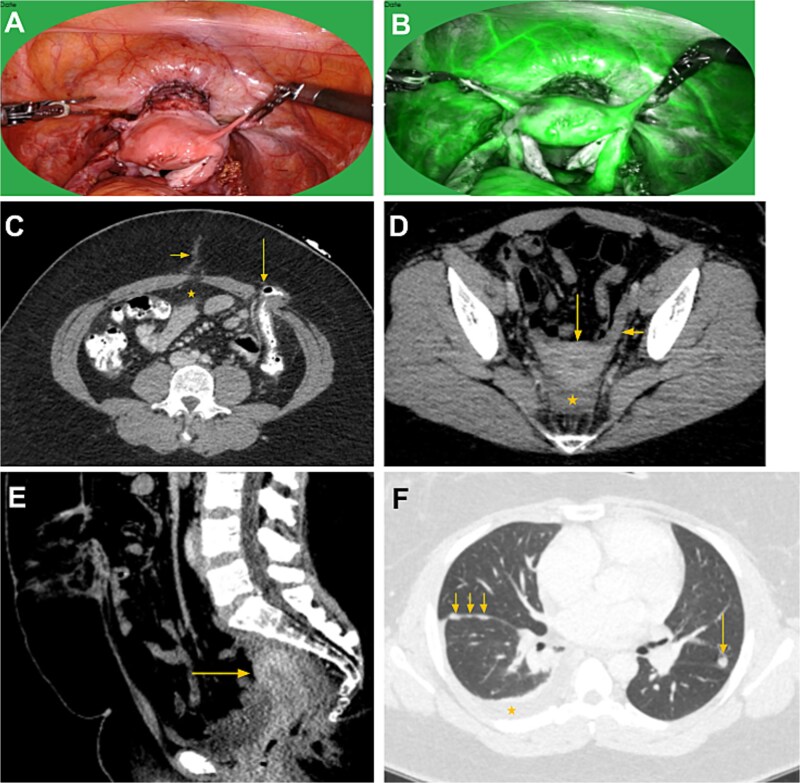
Intraoperative post reposition images (A and B) followed by post-reposition CT abdomen, pelvis, and chest. (C) Axial CT image at the level of the umbilicus shows minimal postsurgical stranding at the umbilical canal (short arrow). The previously transposed uterus is no longer present in the anterior midline abdomen (*). A left abdominal wall end colostomy is noted (long arrow), status post abdominoperineal resection. (D, E) Axial and sagittal pelvic images demonstrate the uterus (long arrows) repositioned in its anatomical location. The left utero-ovarian ligament is visualized (short arrow in D). A small amount of free fluid is seen in the abdominoperineal resection bed (* in E). Ovaries (not shown) have also been repositioned to their anatomical locations. (F) CT chest performed 2 months post-reposition demonstrates metastatic disease to the lungs and right pleura, including a right pleural effusion (*), right fissural nodularity (short arrows), and a left lung nodule (long arrow).

In March 2025, following oncologic clearance, the patient underwent robotic-assisted uterovaginal anastomosis with uterine and ovarian take-down. The procedure was performed in collaboration with colorectal surgery, which simultaneously carried out a robotic-assisted abdominoperineal resection and end colostomy (Fig. 3A–C).

Intraoperative findings: The uterus and adnexa appeared well perfused with preserved vascular pedicles. ICG angiography confirmed robust uterine perfusion. Adhesiolysis was performed to mobilize the uterus from the abdominal wall. The neocervical canal was excised, and an uterovaginal anastomosis was fashioned to re-establish continuity.

Estimated blood loss: 100 ml. No intraoperative complications occurred.

Postoperative course: Recovery was prolonged by ileus and constipation, managed conservatively. She was discharged on postoperative day 10.

### Pathology

Rectosigmoidectomy specimen:

- Invasive moderate-to-poorly differentiated mucinous adenocarcinoma, 3.0 cm.

- 1 of 11 lymph nodes positive for metastatic carcinoma.

### Follow-up

The patient resumed menstruation via the native cervix following UR. Several months later, she developed recurrence (Fig. 3D):

- Pleural effusion: Malignant cytology confirming recurrent disease.

- Vulvar lesions: Resected for palliation; pathology confirmed metastatic recurrence.

She is currently receiving systemic chemotherapy.

## Discussion

This case demonstrates the technical feasibility of complete robotic UT followed by successful robotic uterovaginal anastomosis and UR. To our knowledge, this is the first reported U.S. case of a robotic UT-UR sequence and among the earliest globally [8, 9]. UT is an evolving fertility-preserving strategy designed to protect both ovarian and uterine function in women undergoing pelvic radiation. Unlike traditional ovarian transposition, UT seeks to preserve the uterus itself as a future gestational organ, offering unique potential for complete fertility preservation [1–3]. However, while the surgical technique has matured, long-term oncologic and reproductive outcomes remain incompletely characterized.

Oncologic safety: Oncologic treatment was uninterrupted, though pathology revealed adverse features (nodal metastasis), contributing to eventual recurrence [10]. These findings underscore that while fertility preservation may be surgically achievable, cancer biology remains the primary determinant of prognosis.

Fertility preservation: Despite disease progression, the uterus remained viable post-UR, with resumption of menstruation, supporting the potential for future gestation in similar patients [4–7].

Comparison with literature: Ribeiro *et al.* first described laparoscopic UT in 2017 [3]. Subsequent reports have documented preserved menstruation and isolated pregnancies. Moretti-Marques *et al.* reported a live birth after UT in cervical cancer [4–7]. Gornet *et al.* described a robotic UT with UR in a patient with fibroids and rectal cancer [9] The present case expands on these prior studies by demonstrating a complete robotic UT–UR cycle performed concurrently with radical oncologic resection [1, 8, 9, 11].

Given the investigational nature of UT, ethical considerations remain paramount [2, 12]. Patients must be counseled on uncertain reproductive outcomes, possible ischemic or anastomotic complications, and the possibility of recurrence limiting future fertility. For oncologic teams, this procedure requires close integration with tumor boards and reproductive specialists to ensure that fertility goals never supersede curative intent.

This case underscores the technical feasibility of performing a complete cycle of UT and robotic uterovaginal anastomosis with UR. However, it also highlights a critical reality: feasibility does not equate to oncologic success. The patient ultimately experienced systemic recurrence, which limits the reproductive potential of the preserved uterus and emphasizes that oncologic prognosis remains the primary driver of long-term outcomes [10, 12].

Yet, the adverse pathologic findings in this case—nodal involvement—as well as the aggressive biology illustrates the difficulty of selecting patients who might truly benefit. Even when uterine viability is maintained, recurrence may render fertility preservation moot [10, 12].

This case therefore reinforces that patient selection must be individualized and highly cautious. Candidates should be reproductive-aged women with a strong desire for future fertility, favorable oncologic profiles, and no clear indicators of systemic disease. Robust informed consent is essential, with emphasis on the experimental nature of UT, its uncertain fertility outcomes, and the possibility that recurrence could negate its benefits [2, 4].

Several reports describe successful pregnancies following UT, including the first live birth reported by Ribeiro [5–7]. However, successful pregnancies remain rare and oncologic follow-up is limited. Prospective registries and larger series are urgently needed to clarify whether UT compromises cancer control, and to define which subsets of patients are most likely to achieve meaningful reproductive benefit [2, 5–9].

## Conclusion

Robotic UT followed by uterovaginal anastomosis and UR is technically feasible and can be successfully integrated with complex oncologic surgery. However, this case illustrates that surgical success alone is not sufficient—systemic recurrence ultimately prevented realization of fertility potential.

UT represents a promising but experimental fertility-preserving innovation. Until larger series and prospective registries are available, its use should remain limited to specialized centers with expertise in both minimally invasive and oncologic surgery. Careful patient selection, rigorous counseling, and close multidisciplinary collaboration are essential. Future research must focus on long-term oncologic safety, uterine function, and live-birth outcomes to ensure that the promise of UT does not come at the expense of cancer control.
